# Enriching ultra-high risk for psychosis cohorts based on accumulated exposure to environmental risk factors for psychotic disorders

**DOI:** 10.1017/S0033291724002551

**Published:** 2024-11

**Authors:** Brian O'Donoghue, Dominic Oliver, Hellen Geros, Holly Sizer, Andrew Thompson, Patrick McGorry, Barnaby Nelson

**Affiliations:** 1 Department of Psychiatry, University College Dublin, Dublin, Ireland; 2 Department of Psychiatry, Royal College of Surgeons, Dublin, Ireland; 3 Orygen, Parkville, Melbourne, VIC 3052, Australia; 4 Centre for Youth Mental Health, University of Melbourne, Melbourne, Australia; 5 Department of Psychiatry, University of Oxford, Oxford, UK; 6 NIHR Oxford Health Biomedical Research Centre, Oxford, UK; 7 OPEN Early Detection Service, Oxford Health NHS Foundation Trust, Oxford, UK

**Keywords:** at-risk mental state, clinical high risk, environment, prediction, psychosis

## Abstract

**Background and hypothesis:**

Transition to psychosis rates within ultra-high risk (UHR) services have been declining. It may be possible to ‘enrich’ UHR cohorts based on the environmental characteristics seen more commonly in first-episode psychosis cohorts. This study aimed to determine whether transition rates varied according to the accumulated exposure to environmental risk factors at the individual (migrant status, asylum seeker/refugee status, indigenous population, cannabis/methamphetamine use), family (family history or parental separation), and neighborhood (population density, social deprivation, and fragmentation) level.

**Methods:**

The study included UHR people aged 15–24 who attended the PACE clinic from 2012 to 2016. Cox proportional hazards models (frequentist and Bayesian) were used to assess the association between individual and accumulated factors and transition to psychosis. UHR status and transition was determined using the CAARMS. Benjamini–Hochberg was used to correct for multiple comparisons in frequentist analyses.

**Results:**

Of the 461 young people included, 55.5% were female and median follow-up was 307 days (IQR: 188–557) and 17.6% (*n* = 81) transitioned to a psychotic disorder. The proportion who transitioned increased incrementally according to the number of individual-level risk factors present (HR = 1.51, 95% CIs 1.19–1.93, *p* < 0.001, *p*_corr_ = 0.01). The number of family- and neighborhood-level exposures did not increase transition risk (*p* > 0.05). Cannabis use was the only specific risk factor significantly associated with transition (HR = 1.89, 95% CIs 1.22–2.93, *p*_corr_ = 0.03, BF = 6.74).

**Conclusions:**

There is a dose–response relationship between exposure to individual-level psychosis-related environmental risk factors and transition risk in UHR patients. If replicated, this could be incorporated into a novel approach to identifying the highest-risk individuals within clinical services.

## Introduction

The introduction of criteria for the ultra-high risk (UHR)/clinical high risk (CHR)/at risk mental state (ARMS) for psychosis led to the ability to identify the putative prodromal stages of psychotic disorders and also to develop and evaluate potential interventions to either prevent or delay the onset of a full threshold psychotic disorder (Mei et al., 2021; van der Gaag et al., 2013). While there are different criteria and instruments used to determine those at UHR for psychosis (the term which will be used from here forth), there are broad similarities amongst these methods that consist of the presence of positive psychotic symptoms, either under the threshold of intensity or duration for a psychotic disorder, poor functioning, schizotypy, and a positive family history in a first-degree relative (Miller et al., 1999; Yung et al., 2005). Other criteria include that the individual is help-seeking and within a certain age range (early adolescence to young adulthood).

However, it is now recognized that UHR cohorts do not mirror that of first-episode psychosis (FEP) cohorts and individuals who transition from a UHR service differ in demographic and clinical characteristics to those who enter via an FEP service directly (Hagler et al., 2023). In a typical FEP cohort, there is a preponderance of certain environmental characteristics, including social deprivation (O'Donoghue, Roche, & Lane, 2016), ethnicity, and migration, including second-generation migrants (Selten, van der Ven, & Termorshuizen, 2020) and Indigenous populations (Jewell & Mitchell, 2023), substance use such as cannabis or amphetamines (McKetin et al., 2019; Robinson et al., 2023) and urbanicity and high population density (Kelly et al., 2010). However, in UHR cohorts, it has been found that some of these factors are under-represented, as there can be a higher proportion of native-born populations (Geros et al., 2020; Moore et al., 2021) and those living in more affluent areas (Moore et al., 2023). Although this is not consistently reported, as in the UK, risk in FEP and UHR cohorts was associated with the same neighborhood characteristics, specifically of single-parent households, ethnicity, and deprivation (Kirkbride et al., 2015).

There are potential explanations for these differences in the characteristics of UHR and FEP cohorts, such as help-seeking behaviors, referral pathways, and biases in research participation (Hagler et al., 2023; O'Donoghue, Polari, McGorry, & Nelson, 2022). However, it is worth considering whether the characteristics of UHR cohorts need to mirror that of FEP cohorts. The necessity for these cohorts to be similar is related to the time point at which the environmental factors could exert an influence on the risk of developing a full-threshold psychotic disorder. It may be that an environmental risk factor increases the risk of developing sub-threshold psychotic symptoms and thereby meeting criteria for the UHR state and then other factors are associated with the risk for transition from the UHR state to a full threshold disorder. In this case, the characteristics of the UHR cohort should be similar to that of the FEP cohort. Alternatively, it is possible that an environmental factor acts more proximally in the transition to full threshold psychosis and therefore the environmental risk factors would not influence the risk of being UHR. In this scenario, the environmental risk factors would be associated with an increased risk of transition and the UHR cohorts would not necessarily have to reflect that of the FEP cohort. Additionally, as there is a sub-group of people with an FEP who do not have a prodrome, estimated to be approximately 22% (Benrimoh et al., 2024), it may be that this group has the preponderance of environmental risk factors and if this was the case, then UHR cohorts would also not mirror FEP cohorts.

Another issue in relation to the UHR state is that the transition rates have declined since the initial establishment of these services. Over 40% of UHR individuals transitioned to a full-threshold psychotic disorder in the initial studies in the mid-1990s, and then there has been a steady decline in rates to approximately 15% (Hartmann et al., 2016). Furthermore, there has been an argument against the concept of specific UHR services due to one study in the UK finding that only a small proportion of individuals (4.1%) in their first-episode services initially attended the attached UHR services (Ajnakina et al., 2017). In another study in Melbourne, it was found that 13.7% of those with an FEP first attended a UHR service and another 7.6% attended a different youth early intervention service (Burke et al., 2022). While there has been a call to abandon these services altogether based on these findings (Ajnakina, David, & Murray, 2019), it could also be considered that these findings are a result of the UHR clinics having been effective and preventing people from developing a full threshold psychotic disorder. Considering both of these viewpoints, there would still be merit in testing the hypothesis that UHR cohorts could be ‘enriched’ with people at even greater risk for transitioning to a psychotic disorder based on the presence of established environmental risk factors for psychotic disorders by bringing the characteristics of the UHR cohort to more closely resemble that of the FEP cohort. This can still be done alongside the development of youth mental health services that are easy to access and are transdiagnostic. This may be a more actionable goal than basing enrichment on biological risk factors, which are less well established (Sanfelici, Dwyer, Antonucci, & Koutsouleris, 2020). A proof of principle study by Padmanabhan, Shah, Tandon, and Keshavan (2017) found that exposure to an accumulated number of environmental risk factors increased the risk for young people with a relative with a diagnosis of schizophrenia to subsequently develop a full threshold psychotic disorder (Padmanabhan et al., 2017). The environmental risk factors examined included cannabis use, urbanity, season of birth, paternal age, obstetrical complications, and childhood adversity. The authors commented that these findings lend support for the ‘multiple hit’ hypothesis for the development of psychosis but their findings were also limited to young people at familial high risk and they identified that it could be applicable to a broader group who are at risk for developing psychosis.

The current study aimed to determine whether the proportion of individuals within a UHR cohort who transition to an FEP varied according to the presence of accumulated environmental risk factors for psychotic disorders. It was hypothesized that the transition rates would be lowest in the sub-group of UHR young people with no environmental risk factors present and that the transition rate would increase incrementally with a higher accumulated number of environmental risk factors.

## Methodology

### Setting

The Personal Assessment and Crisis Evaluation (PACE) service is a specialized outpatient clinic of Orygen, a public mental health service for young people aged 15–24 years who reside in the north-western region of Melbourne. The PACE clinic is attended by young people identified as UHR for psychosis, as operationalized using the CAARMS criteria (Yung et al., 2005). There is an open community source of referrals and they typically come from general practitioners, counsellors, and community health services, as well as by self-referrals. An individual identified as UHR will receive care over a period spanning between 9 and 12 months, but can receive care for a maximum of 2 years or until their 18th birthday, whichever is longer.

### Participants

This study included all young people meeting UHR criteria who attended the PACE service between 1st January 2012 and 31st December 2016 and all consecutive cases for this period were included in the study, thereby minimizing selection bias.

### Study design

This was an observational cohort study. Relevant information was recorded prospectively in the clinical file by clinicians and extracted retrospectively by researchers for this study. Structured assessments using validated instruments were conducted at baseline and other timepoints by trained clinicians and data were extracted from these assessments. Client files and electronic medical records were used to access demographic data.

### Instruments

The CAARMS is a valid and reliable instrument that can determine the presence of the at-risk mental state (Yung et al., 2005) and the criteria for the UHR state is provided in online Supplementary Table S1. The CAARMS was completed at baseline to determine eligibility to enter the UHR clinic. As this was a clinical service, the young person was reviewed regularly by their case manager and doctor, usually weekly initially and then forthnightly depending upon the clinical indication. If there was a concern that the young person's psychotic symptoms had worsened, then a CAARMS was also used to determine if an individual had transitioned to full-threshold psychosis, as it provides clear criteria for the presence of a psychotic disorder. Therefore, the follow-up CAARMS were not completed at a fixed timepoint, but rather when indicated. The Global Assessment of Functioning was used to determine the level of functioning. It is scored from 0 to 100 with higher score indicating higher levels of functioning (First, Spitzer, & Williams, 1995).

### Environmental risk factors

The environmental risk factors were categorized according to three levels, specifically individual, family, and neighborhood levels. Individual-level factors included migrant status, being an asylum seeker or refugee, identifying as Aboriginal or Torres Strait Islander, current or past cannabis use, current or past amphetamine use (5 factors). The family-level factors were a family history of a psychotic disorder in a first-degree relative, a second-degree relative and a history of parental separation (3 factors). The neighborhood-level factors included residing in the most socially deprived neighborhoods, the most densely populated neighborhoods or those with the highest level of social deprivation (3 factors). These environmental risk factors were included based on availability, and other environmental risk factors, such as obstetrical complications and trauma, were not available.

### Classification and definitions of environmental risk factors

At the time of first registration with the service, individuals were asked about their country of birth and whether they identified as Aboriginal or Torres Strait Islander. A first-generation migrant was defined as an individual who was born in another country other than Australia and moved to Australia after birth. As we did not have data pertaining to the place of birth of both parents, we were unable to determine whether an individual was a second-generation migrant. Individuals were asked whether they were asylum seekers or had been granted refugee status.

Social deprivation was determined using the Index of Relative Socio-Economic Disadvantage from the Australian Bureau of Statistics. Social fragmentation is a composite measure composed of four census variables: the percentage of single-person households, dwellings rented, persons having lived at a different address 1-year prior, and (socially defined) unmarried persons. The population density was calculated from the total population residing within a postcode divided by the area of the postcode. A more detailed description of how each of the neighborhood-level characteristics was determined is provided in online Supplementary Table S1.

### Statistical analysis

The cumulative risk of psychosis onset in this sample was described through the Kaplan–Meier failure function (1 – survival) (Kaplan & Meier, 1958). Missing data were assumed to be missing at random through visualization with the ‘naniar’ (version 0.6.1) package (Tierney & Cook, 2023) (online Supplementary eFig. 1). Missing data were imputed using multivariate imputation by chained equations across 50 iterations, implemented in the ‘mice’ package (version 3.15.0) (van Buuren & Groothuis-Oudshoorn, 2011), using logistic regression imputation, ‘polyreg’ for categorical and predictive mean matching ‘pmm’ for continuous variables, pooled using Rubin's rule (Rubin, 1987). All environmental risk factors and time-to-event data were included in the imputation model with no auxiliary data. All analyses were conducted using the imputed dataset.

Two sets of analyses were conducted. First, the proportions who transitioned according to the accumulated exposure of factors in each category were determined. For example, the proportion who transitioned according to the presence of zero up to six individual-level risk factors was determined. Second, the transition rates according to the exposure of all the factors across individual, family and neighborhood levels in an additive exposome score were determined (Pries, Erzin, Rutten, van Os, & Guloksuz, 2021).

The proportion who transitioned to a full threshold psychotic disorder was the outcome of interest for this study and therefore the percentage in each cohort who transitioned was presented, as it is a meaningful, clinically relevant outcome. However, there was a varying length of follow-up for individuals included in this study and also transition was an event that could occur at any point. Therefore, Cox proportional hazards models were used to test the association between the number of exposures within each category as a continuous variable and individual exposures as binary variables using the ‘survival’ package (version 3.5-0) in the software package R. Hazard ratios with 95% confidence intervals were determined and presented alongside the proportions. The date of entry to the cohort was the date of the initial assessment, representing the time of presentation. The date of exit was either the date of transition, determined by the CAARMS, or the time of the last assessment prior to either discharge or disengagement from the clinical service.

These analyses were complemented by Bayesian inference for Cox proportional hazard models to primarily estimate certainty of evidence for the null hypothesis using the ‘baymedr’ package (version 0.1.1.9). We report a Bayes Factor (BF), which relates to the strength of the evidence for the investigated factor to be associated with transition. We used Jeffreys' classification scheme to interpret BF (Jeffreys, 1998), with values less than 1 provide weak (0.333 < BF < 1) and moderate (0.1 < BF < 0.333) evidence for the null hypothesis (no increase in transition risk with exposure to risk factor). Meanwhile, BF values greater than 1 provide weak (1 < BF < 3) and moderate (3 < BF < 10) evidence for the alternative hypothesis (increase in transition risk with exposure to risk factor).

Frequentist statistics were adjusted for multiple comparisons using the Benjamini–Hochberg correction. Analyses were conducted with R version 4.2.2. The threshold for statistical significance was *p* < 0.05.

### Ethics

This project received ethical approval from the Melbourne Health HREC (QA2016141).

## Results

### Description of participants

A total of 461 young people identified as UHR attended the PACE clinic during the 5-year study period. Of these, 55.5% (*N* = 256) were female and the mean age was 18.4 (±2.8) years. The mean level of functioning according to the GAF was 52.6 (±9.4). The majority were not married (95.2%, *N* = 439) and 30.2% (*N* = 139) were not in employment, education, or training. A total of 13.7% (*N* = 63) were first-generation migrants, and 2.8% (*N* = 13) identified as Aboriginal or Torres Strait Islanders. Cannabis use was present in 31.5% (*N* = 145) of the cohort and 13.2% (*N* = 61) were using methamphetamines. In total, 276 (4.6%) data points were missing and imputed using MICE. The demographic and clinical characteristics of the cohort are presented in Table 1.

**Table 1.** Demographic and clinical characteristics

	*N* = 461
	Mean (±s.d.)
Age	18.4 (2.8)
Sex	% (*n*)
Male	44.5 (205)
Female	55.5 (256)
Marital status – % never married	95.2 (439)
Living arrangements – % with parents	67.8 (746)
Not in employment, education or training	30.2 (139)
Family history of psychosis	
First-degree relative	21.7 (100)
Second-degree relative	13.2 (61)
Cultural background	
Migrant	13.7 (63)
Asylum seeker or refugee	1.5 (7)
Aboriginal or Torres Strait Islander	2.8 (13)
Substance misuse	
Cannabis	31.5 (145)
Amphetamine	13.2 (61)
UHR criteria present	
Attenuated Psychotic Symptoms (APS)	86.1 (397)
Brief Limited Psychotic Symptoms (BLIPS)	3.9 (18)
Family history and poor functioning	19.5 (90)
Functioning	Mean (±s.d.)
Global Assessment of Functioning (GAF)	52.6 (9.4)

### Proportion who transitioned to a full threshold psychotic disorder

**Figure 1.**
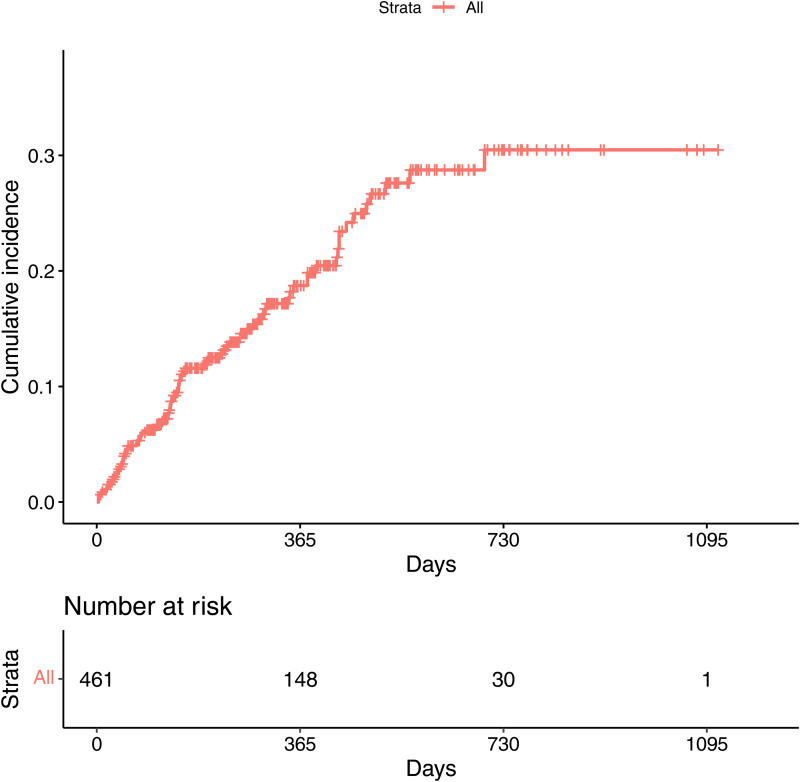
Kaplan–Meier survival curve showing cumulative probability of transition to psychosis with Greenwood 95% confidence intervals. The number at risk at each timepoint presented below refers to the number of individuals who are no longer being followed-up, either due to transition to psychosis or censoring.

The median follow-up was 307 days (IQR = 188–557). During this time, 17.6% (*N* = 81) were known to have transitioned to a full threshold psychotic disorder. The median transition time was 142 days (IQR = 31–349). The cumulative incidence of psychosis was 0.12 (95% CIs 0.08–0.15, 327 still at risk) at 6 months, 0.19 (95% CIs 0.14–0.23, 155 still at risk) at 12 months, 0.28 (95% CIs 0.22–0.34, 78 still at risk) at 18 months and 0.305 (95% CIs 0.230–0.373, 30 still at risk) at 24 months (Fig. 1).

### Transition according to individual-level risk factors

A total of 54.2% (*N* = 250) UHR young people had no individual-level risk factors present and of these, 14.0% (*N* = 35) transitioned to a full threshold psychotic disorder. One hundred and thirty-seven UHR young people had one individual-level risk factor present and 19.7% (*N* = 27) transitioned. Sixty-three UHR young people had two individual-level risk factor present and 20.6% (*N* = 13) transitioned. Eleven UHR young people had three individual-level risk factor present and 54.5% (*N* = 6) transitioned. The proportion of UHR young people who transitioned increased incrementally according to the number of individual-level risk factors present (HR = 1.51, 95% CIs 1.19–1.93, *p* < 0.001, *p*_corr_ = 0.01).

**Figure 2.**
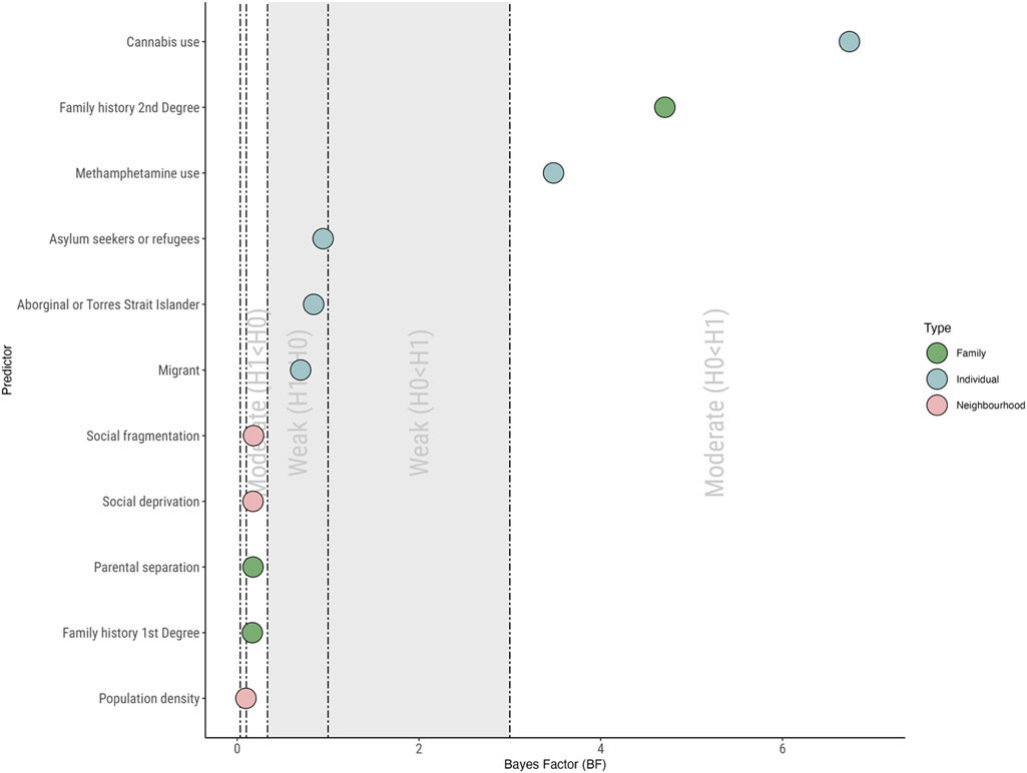
Results of Bayesian inference of Cox proportional hazard models for each environmental risk factor. 0.1 < Bayes Factor (BF) < 0.333, moderate evidence for the null hypothesis (H0; no increase in transition risk with exposure to risk factor); 0.333 < BF < 1, weak evidence for the null hypothesis (H0; no increase in transition risk with exposure to risk factor); 1 < BF < 3, weak evidence for the alternative hypothesis (H1; increased transition risk with exposure to risk factor); 3 < BF < 10, moderate evidence for the null hypothesis (H1; increased transition risk with exposure to risk factor).

Cannabis use (HR = 1.89, 95% CIs 1.22–2.93, *p* = 0.004, *p*_corr_ = 0.03, BF = 6.74) and methamphetamine use (HR = 2.02, 95% CIs 1.15–3.56, *p* = 0.01, BF = 3.48) significantly increased transition risk and presented moderate evidence for the alternative hypothesis (Fig. 2). However, methamphetamine use was not statistically significant following correction for multiple comparisons (*p*_corr_ = 0.07). No other individual-level risk factors significantly increased transition risk alone (*p* > 0.05) and all presented weak evidence for the null hypothesis (BF = 0.70–0.95, Fig. 2).

### Transition according to family-level risk factors

A total of 28.2% (*N* = 130) had no family-level risk factors present and of these, 17.7% (*N* = 23) transitioned to a full threshold psychotic disorder. Two hundred and twenty-six UHR young people had one family-level risk factor present and 18.6% (*N* = 42) transitioned. Ninety-one UHR young people had two family-level risk factors present and 16.5% (*N* = 15) transitioned. Fourteen UHR young people had three family-level risk factors present and 7.1% (*N* = 1) transitioned. There was no significant association between the number of family-level risk factors present and transition (HR = 0.97, 95% CIs 0.73–1.29, *p* = 0.82).

Having family history of psychosis in a second-degree relative significantly increased transition risk (HR = 1.95, 95% CIs 1.13–3.38, *p* = 0.017) and presented moderate evidence for the alternative hypothesis (BF = 4.71, Fig. 2). This was no longer significant following correction for multiple comparisons (*p*_corr_ = 0.07). No other family-level risk factors significantly increased transition risk alone (*p* > 0.05). Family history of psychosis in a first-degree relative (BF = 0.17) and parental separation (BF = 0.17) both presented moderate evidence for the null hypothesis (Fig. 2).

### Transition according to neighborhood-level risk factors

A total of 41.2% (*n* = 190) did not have an exposure to a neighborhood-level risk factor and of these, 20.0% (*n* = 38) transitioned to a full threshold psychotic disorder. Ninety-nine UHR young people had one neighborhood-level risk factor present and 16.2% (*N* = 16) transitioned. One hundred and forty-one UHR young people had two neighborhood-level risk factors present and 16.2% (*N* = 23) transitioned. Thirty-one UHR young people had three neighborhood-level risk factors present and 12.9% (*N* = 4) transitioned. There was no significant association between the number of neighborhood-level risk factors present and transition (HR = 0.87, 95% CIs 0.70–1.09, *p* = 0.24).

No neighborhood-level risk factors significantly increased transition alone (*p* > 0.05). Moderate evidence for the null hypothesis was found for social deprivation (BF = 0.17) and social fragmentation (BF = 0.18) (Fig. 2). Strong evidence for the null hypothesis was found for population density (BF = 0.096) (Fig. 2).

### Transition according to total additive exposome score

When all environmental risk factors were examined collectively as an additive exposome score, there were 80 UHR young people who did not have any of these factors present and of these, 9.09% (*n* = 3) transitioned to a full threshold disorder. The proportion of each group that transitioned to a full threshold psychotic disorder according to the number of environmental risk factors present are displayed in Table 2. There was no significant association between additive exposome score and transition (HR = 1.07, 95% CIs 0.92–1.23, *p* = 0.39).

**Table 2.** Proportion and hazard ratios for transition according to the number of environmental risk factors

	*N*	Transitioned	Did not transition	Hazard ratio	95% CI	*p*
Individual-level environmental risk factors						
None present	27	11.1 (3)	88.9 (24)	Ref		
At least one present	434	18.0 (78)	82.0 (356)	1.24	0.39–3.94	0.715
At least two present	423	18.0 (76)	82.0 (347)	1.23	0.39–3.91	0.725
At least three present	149	22.1 (33)	77.9 (116)	1.61	0.49–5.26	0.433
At least four present	23	26.1 (6)	73.9 (17)	1.25	0.29–5.38	0.761
Five risk factors present	3	33.3 (1)	66.7 (2)	3.77	0.62–23.0	0.151
Family-level environmental risk factors						
None present	219	18.7 (41)	81.3 (178)	Ref		
At least one present	242	16.5 (40)	83.5 (202)	0.82	0.52–1.29	0.381
At least two present	91	14.3 (13)	85.7 (78)	0.67	0.34–1.31	0.242
Three present	11	9.1 (1)	90.9 (10)	0.50	0.07–3.66	0.496
Neighborhood-level environmental risk factors						
None present	201	19.4 (39)	80.6 (162)	Ref		
At least one present	260	16.2 (42)	83.8 (218)	0.78	0.49–1.23	0.286
At least two present	163	16.0 (26)	84.0 (137)	0.77	0.46–1.29	0.316
At least three present	27	14.8 (4)	85.2 (23)	0.47	0.11–1.96	0.302
All environmental risk factors						
None present	27	11.1 (3)	88.9 (24)	Ref		
At least one present	434	18.0 (78)	82.0 (356)	1.24	0.39–3.94	0.715
At least two present	432	18.1 (78)	81.9 (354)	1.25	0.39–3.95	0.710
At least three present	377	18.8 (71)	81.2 (306)	1.28	0.40–4.07	0.677
At least four present	281	18.9 (53)	81.1 (228)	1.26	0.39–4.06	0.694
At least five present	158	16.5 (26)	83.5 (132)	1.04	0.31–3.45	0.955
At least six present	76	11.8 (9)	88.2 (67)	0.84	0.22–3.19	0.802
At least seven present	22	9.1 (2)	90.9 (20)	0.33	0.03–3.15	0.334
Eight present	4	0 (0)	100 (4)	0.04	0.01–5759	0.580

## Discussion

### Summary of findings

The main finding of this study is that the proportion of UHR young people who transitioned to a full threshold psychotic disorder increased incrementally when the number of individual-level environmental risk factors increased, including being a first-generation migrant, an asylum seeker or refugee, Indigenous population, a history of cannabis or methamphetamine use. However, this was not found with the accumulated number of family or neighborhood-level risk factors present or when all of the environmental risk factors were examined collectively. These results indicate that there is potential to identify the highest risk group within the UHR cohorts for psychosis risk using individual-level environmental risk factors, but not family- and neighborhood-level risk factors.

### Comparison to previous literature

It has been found that an accumulated number of environmental risk factors, particularly individual factors such as cannabis use, is associated with an earlier age of onset for FEP (O'Donoghue et al., 2015a). Therefore, it is possible that the findings of the current study could be explained by an earlier age of transition, as opposed to an increase in risk for transition, especially considering that the follow-up period in this study was relatively short, at approximately 1 year and the cohort were young (mean age = 18 years). While a history of cannabis use is not associated with an increased risk of transition to a full threshold psychotic disorder (Farris, Shakeel, & Addington, 2020), current cannabis abuse is associated with an increased risk of transition to psychosis (Kraan et al., 2016). Furthermore, the relationship may not be directly causal, as there is an overlap between the genetic risk for both cannabis use and psychotic disorders (Cheng et al., 2023).

Previous studies have attempted to determine the risk for developing psychosis based on environmental risk factors through an exposome score. Our results have shown no significant association between transition to psychosis and an additive exposome score. Another approach is through weighting risk factors according to their meta-analytic effect sizes such as in the ‘Maudsley Environmental Risk Score for Psychosis’ (Vassos et al., 2020), Psychosis Polyrisk Score (PPS) (Oliver et al., 2020; Oliver, Radua, Reichenberg, Uher, & Fusar-Poli, 2019), and the Korea-Polyenvironmental risk score for psychosis (Jeon et al., 2022). There is some evidence to suggest that using meta-analytic weightings may not perform as well as using either an additive score, as we used in this study, and many of the risk factors do not have meta-analytic estimates. A more sophisticated modelling approach that allows to take the correlations between risk factors into account may perform better but would benefit from a larger sample size and a greater range of risk factors (Pries et al., 2019).

### Clinical implications and further research

The findings of this research indicate that there is potential for UHR cohorts to be enriched by incorporating the presence of individual-level environmental risk factors alongside the current criteria based on symptoms and functioning into the criteria for entry to services. There was a clear incremental increase in the proportion of young people who transitioned to a full threshold psychotic disorder as the number of accumulated individual-level environmental risk factors increased, with 18% transitioning when there were one or two factors present and up to 33% when five factors were present. However, the numbers were particularly small in the later groups which had a higher number of individual risk factors present. There were a number of environmental risk factors that were not present in this analysis, such as childhood trauma and prenatal and perinatal insults which are established risk factors for developing a psychotic disorder (Davies et al., 2020; Sideli et al., 2020). Therefore, the findings of this study would need to be replicated in a larger UHR prospective study, such as the current Accelerating Medicines Partnership Schizophrenia (AMP-SCZ) study (Wannan et al., 2024), which will have sufficient numbers in the groups with higher numbers of accumulated individual risk factors. If replicated, then it would be warranted to test this hypothesis in a prospective study and it could be determined whether the inclusion of accumulated environmental risk factors could improve the prediction to a psychotic disorder.

The ‘prevention paradox’ states that the majority of cases of a disorder or disease will come from the far larger proportion of the population with moderate or slightly above average risk (Rose, 1992). The crux of this argument is that more of a disorder could be prevented by shifting the mean of the overall population toward a healthier profile, as opposed to targeting the minority who are at very high risk. However, for the objective of preventing the transition to psychosis, it is possible to take both approaches (Yung et al., 2021). For young people presenting with mental health problems it is warranted to provide free, widely available, and easily accessible youth mental health services that provide early interventions for high prevalence mental health difficulties and disorders. These primary care services are provided by headspace in Australia (Rickwood et al., 2019) and similar services are provided in places such as Canada, the UK, and also Ireland (Malla et al., 2016). However, more specialized services, such as early intervention for psychosis services, are required alongside these primary care services. Within these services, the enriched UHR cohorts could receive assessment and evidence-based treatments.

It is a curious finding that the presence of family and neighborhood factors was not associated with a higher rate of transition, as these are established risk factors for a psychotic disorder (Gottesman & Erlenmeyer-Kimling, 2001). It is possible that these risk factors have a role earlier in the development of a psychotic disorder, in that they may increase the risk of individuals developing the at-risk mental state but they are not involved in the progression further to a full threshold psychotic disorder. This is supported by the previous finding that neighborhood social deprivation was not associated with an increased risk of transition (Moore et al., 2023; O'Donoghue et al., 2015b) and there was a trend for individuals identified as UHR to be from the more socially deprived neighborhoods (Moore et al., 2023; O'Donoghue et al., 2015c).

It would be more practical for the findings of this study to assist in the identification of the highest risk individuals within a UHR service. It is an ethical question as to whether it is appropriate to have ethnicity or identifying as Indigenous as part of the entry criteria for a clinical service. On one hand, these populations are at greater risk for developing a psychotic disorder which the UHR services aim to prevent or delay. Therefore, it could be justified to prioritize access to these services for these individuals. However, the practice of prioritizing access to services based on ethnicity, even if the intention is to facilitate access for high-risk groups, may result in perceived or real discrimination toward other ethnicities. In addition, it would be challenging to do this in practice, as different ethnic groups are at risk in different countries (Moore et al., 2021) and the risk may also vary within countries as well. At present, ethnic minorities and migrant groups are often under-represented within UHR services (Moore et al., 2021) and therefore, a first step would be to bring their representatives on a par with the majority ethnicity. Therefore, this approach would involve educational campaigns directed toward ethnic minorities on the early warning signs of psychosis and reducing barriers to care for these populations. Additionally, there has been a call for the underlying social determinants of mental disorders to be addressed with the aim of primary prevention of mental health disorders (Kirkbride et al., 2024). For example, neighborhood factors, trauma, and obstetrical complications are more common in certain ethnic groups in the US, specifically Black and Latino people, who are also at greater risk of psychosis (Anglin et al., 2021). Therefore, if the findings of this study are replicated and found to be robust, addressing each of these social determinants will also accumulatively reduce the risk for psychosis. The findings of this study could also lead to the identification of sub-groups within UHR services at higher risk for transition, who could then be offered additional interventions or longer periods of observation.

### Strengths and limitations

There were a number of strengths to this study, such that we were able to include consecutive cases of young people identified as UHR, thereby avoiding any participation bias. We were also able to examine the influence of a large number of environmental risk factors across different levels of exposure. However, the findings need to be considered within the limitations of the study. First, there were a number of environmental risk factors which we did not have data on, such as obstetrical complications and the experience of traumatic events in childhood. Furthermore, some of the data pertaining to the predictor variables were broad and non-specific, such as the lack of information on the timing and quantity of substance abuse. While the data were recorded prospectively, it was collected retrospectively and this meant that any missing data were irretrievable. Additionally, the number of cases in the exposed group reduced substantially as the number of accumulated environmental risk factors increased and the absolute number of cases that transitioned were low. Finally, the median time to follow-up was less than 1 year and the risk of transition continues for a number of years beyond this.

## Conclusions

The proportion of UHR individuals that transitioned to a full threshold psychotic disorder increased incrementally according to the number of accumulated individual-level environmental risk factors for a psychotic disorder, but not family or neighborhood-level risk factors. If replicated, this novel approach of applying entry criteria to a UHR service based on the presence of individual-level environmental risk factors could be piloted within a clinical service.

## Supporting information

O'Donoghue et al. supplementary material O'Donoghue et al. supplementary material

## Supplementary material

The supplementary material for this article can be found at https://doi.org/10.1017/S0033291724002551.
