# Supplementary material for: Enriching ultra-high risk for psychosis cohorts based on accumulated exposure to environmental risk factors for psychotic disorders
Source: Psychol Med. 2024 Nov 25;54(15):4255–63. doi: 10.1017/S0033291724002551 (PMC11650166; doi:10.1017/S0033291724002551)
Supplement: O'Donoghue et al. supplementary material [file S0033291724002551sup001.docx]

|  |
| --- |
| *Criteria for Ultra-high risk for psychosis from the CAARMS* |
| The criteria for the UHR state from the CAARMS stipulates that, to be identiﬁed as UHR for psychosis, an individual must be help-seeking and experience at least one of the following: (i) Attenuated Positive Psychotic Symptoms (APS): subthreshold symptom intensity or frequency present within the last year; (ii) Brief Limited Intermittent Psychotic Symptoms (BLIPS): full-threshold psychotic symptoms that have lasted no longer than a week in the last year and spontaneously remitted (without treatment); or (iii) Trait Vulnerability for a psychotic disorder: presence of risk factors of schizotypal personality disorder or a ﬁrst-degree relative with a psychotic disorder (Yung et al., 2005). Individuals must also have impaired functioning. |
| *Classification of neighbourhood characteristics* |
| Social deprivation was determined using the Index of Relative Socio-Economic Disadvantage from the Australian Bureau of Statistics and it was divided into quartiles, ranging from affluent to deprived. The level of social deprivation was determined from the neighbourhood level of income, education level, employment, occupation, housing and other measures such as receipt of disability benefits. Social fragmentation is a composite measure composed of four census variables: the percentage of single-person households, dwellings rented, persons having lived at a different address one-year prior, and (socially defined) unmarried persons. The postcodes were arranged into quartiles according to the level of social fragmentation (Congdon, 1996). The population density was calculated from the total population residing within a postcode divided by the area of the postcode and these were also arranged into quartiles. The most socially deprived, fragmented and densely populated quartiles were used to define presence of neighbourhood level risk factors, i.e. living in a postcode that was in the ‘most deprived’ quartile for social deprivation was defined as having this particular neighbourhood level risk factor and this definition was also used for social fragmentation and population density. For each neighbourhood characteristic, postcodes were arranged into quartiles, based on their ranking within the state of Victoria in Australia. This method was deemed more appropriate than ranking each postcode within the catchment area because the catchment area is located within an area of Melbourne with higher social deprivation. Therefore, if postcodes were ranked just within the catchment area, it could potentially result in deprived areas being classified as affluent. |


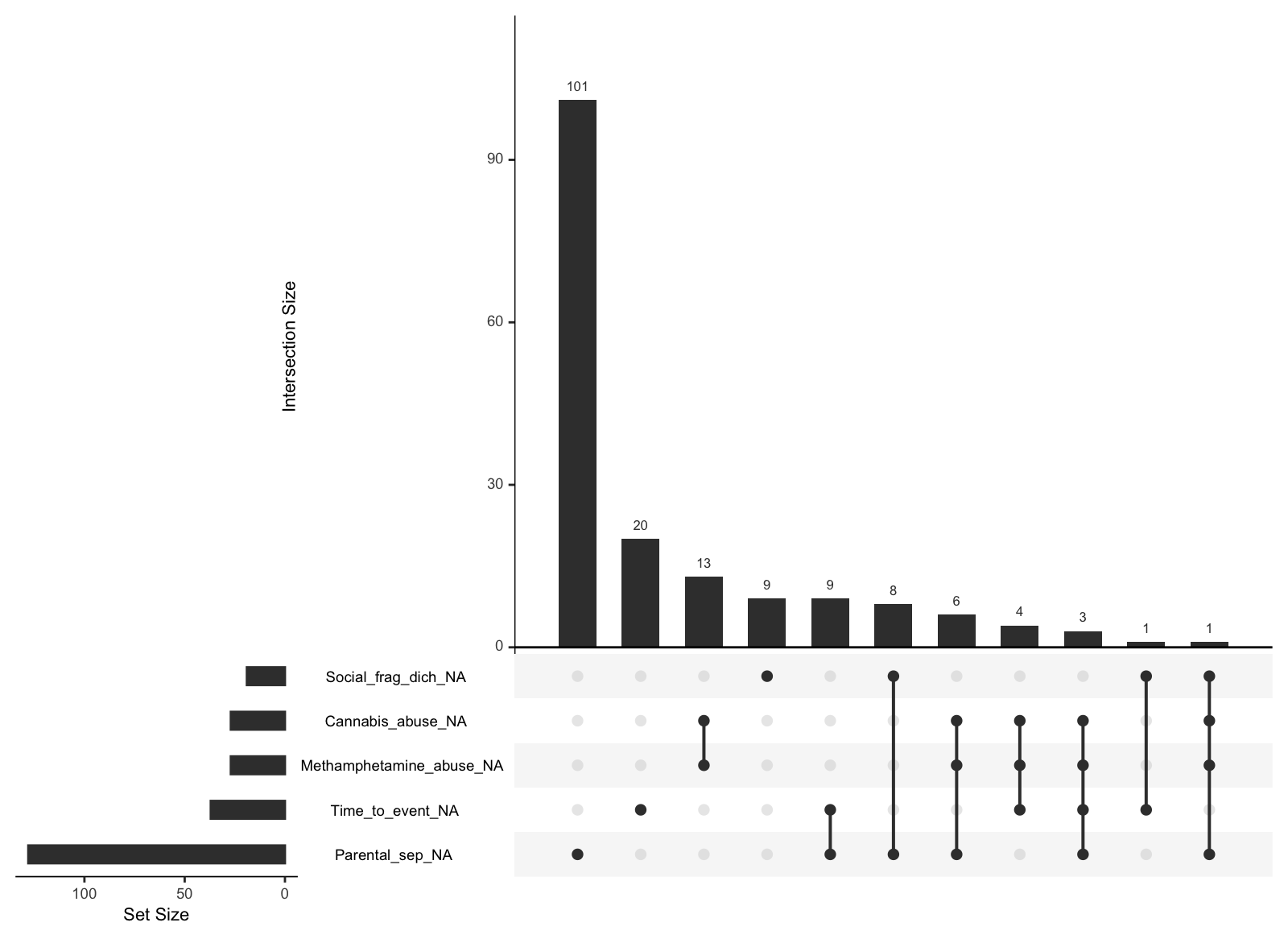


eFigure 1: Missing data assumed to be missing at random through visualisation with the ‘naniar’ (version 0.6.1) package.
